# Short Sleep Duration and Childhood Obesity: Cross-Sectional Analysis in Peru and Patterns in Four Developing Countries

**DOI:** 10.1371/journal.pone.0112433

**Published:** 2014-11-13

**Authors:** Rodrigo M. Carrillo-Larco, Antonio Bernabé-Ortiz, J. Jaime Miranda

**Affiliations:** 1 CRONICAS Center of Excellence in Chronic Diseases, Universidad Peruana Cayetano Heredia, Lima, Peru; 2 Department of Medicine, School of Medicine, Universidad Peruana Cayetano Heredia, Lima, Peru; Medical College of Soochow University, China

## Abstract

**Background:**

We aimed to describe the patterns of nutritional status and sleep duration in children from Ethiopia, India, Peru and Vietnam; to assess the association between short sleep duration and overweight and obesity, and if this was similar among boys and girls in Peru.

**Methods and Findings:**

Analysis of the Young Lives Study, younger cohort, third round. In Ethiopia there were 1,999 observations, 2,011, 2,052 and 2,000 in India, Peru and Vietnam, respectively. Analyses included participants with complete data for sleep duration, BMI, sex and age; missing data: 5.9% (Ethiopia), 4.1% (India), 6.0% (Peru) and 4.5% (Vietnam). Exposure was sleep duration per day: short (<10 hours) versus regular (10–11 hours). Outcome was overweight and obesity. Multivariable analyses were conducted using a hierarchical approach to assess the effect of variables at different levels. Overweight/obesity prevalence was 0.5%/0.2% (Ethiopia), 1.3%/0.3% (India), 6.1%/2.8% (Vietnam), and 15.8%/5.4% (Peru). Only Peruvian data was considered to explore the association between short sleep duration and overweight and obesity, with 1,929 children, aged 7.9±0.3 years, 50.3% boys. Short and regular sleep duration was 41.6% and 55.6%, respectively. Multivariable models showed that obesity was 64% more prevalent among children with short sleep duration, an estimate that lost significance after controlling for individual- and family-related variables (PR: 1.15; 95%CI: 0.81–1.64). Gender was an effect modifier of the association between short sleep duration and overweight (p = 0.030) but not obesity (p = 0.533): the prevalence ratio was greater than one across all the models for boys, yet it was less than one for girls.

**Conclusions:**

Childhood overweight and obesity have different profiles across developing settings. In a sample of children living in resource-limited settings in Peru there is no association between short sleep duration and obesity; the crude association was slightly attenuated by children-related variables but strongly diminished by family-related variables.

## Introduction

The prevalence of childhood obesity has risen in the last two decades; moreover, in the year 2010 approximately 80% of children with overweight/obesity were in developing countries [1]. Childhood obesity is also a concern in developing countries. For example, in the year 2010, Peru reported an overweight and obesity prevalence of 15.5% and 8.9% respectively, for children aged five to nine years [2]. Similarly, the prevalence of overweight, for children aged five to sixteen years, was 6.6% in the year 2005 in India [3]; Ethiopia reported a prevalence of 3.3% in school-aged children [4]. Lastly, in Vietnam there was a prevalence of 6.7% and 2.0% of overweight and obesity, respectively, among children aged eleven to fourteen years old [5].

Sleep duration has emerged as a risk factor for childhood obesity. Short sleep duration has been associated with weight gain, higher body mass index (BMI) values and increased odds of being overweight or obese. This is supported by cross-sectional [6]–[14], as well as longitudinal studies [15]–[21] and meta-analyses [22], [23]; notwithstanding, some authors have not reached this conclusion. Chen et al. reported that children with short sleep duration have 58% higher chances of being obese [22]; others have concluded that short-sleep children have almost twice the odds of being obese, versus their peers with longer sleep duration [23]. Interestingly, it has been reported that this association is not consistent in boys and girls, as some studies have reported this finding only in boys [10], [14].

The association between short sleep duration and overweight or obesity has been mostly studied in developed countries. As several have suggested [9]–[11], [24], [25], these observations may not be fully extrapolated to children in developing countries and studies that include a wider diversity of ethnic groups and socio-demographic variables have been recommended [26]. The Young Lives Study focused explicitly in low-income settings across different world regions and offers an excellent opportunity to address this public health issue. The objective of this study was to describe the patterns of nutritional status and sleep duration of school-aged children in four developing countries, and to assess the association between short sleep duration and overweight and obesity; in addition, we aimed to assess if the association was similar among boys and girls.

## Methods

### Study Design

This is a cross-sectional secondary analysis using data from the Young Lives study [27] conducted in four developing countries: Ethiopia, India, Peru and Vietnam. The Young Lives study began in the year 2002 and continues to date. This study comprises questionnaires on nutrition and health, including anthropometric measurements. Details, such as objectives, rationale, and procedures have been published elsewhere [27]. The data is available online at www.younglives.org.uk.

### Population

The Young Lives study included two longitudinal prospective cohorts, a younger and an older one. At baseline, in the year 2002, the younger cohort recruited children aged 6 to 18 months old: 1,999, 2,011, 2,052 and 2,002 in Ethiopia, India, Peru and Vietnam, respectively (this correspond to the sample size in each country); whereas the older cohort recruited children aged between 7 and 8 years old. We used data from the third round in 2009 of the younger cohorts which had sleep duration data availability. The attrition rate for the younger cohort at the third round was 1.5% for Ethiopia, 1.0% for India, 1.1% for Peru and 0.6% for Vietnam [27].

There were 1,999 participants in Ethiopia's dataset, 2,011 in India's, 2,052 in Peru's and 2,000 in Vietnam's (two subjects were not included because of data inconsistency). Overall, missing values for the main variables –sex, age, sleep duration and BMI– were 5.9% (included subjects: 1,882), 4.1% (included subjects: 1,929), 6.0% (included subjects: 1,929) and 4.5% (included subjects: 1,910) for Ethiopia, India, Peru and Vietnam, respectively; subjects with missing values were excluded from the analysis

### Settings

These countries are considered developing countries, though there are some differences between them. According to the World Bank Peru is an upper middle country, India is a lower middle income country and so is Vietnam, yet Ethiopia is a low income country. Although the study sample in each country is not nationally representative, it is informative of children the right age in low-income settings from different world regions; these populations have not been widely studied in terms of overweight, obesity and sleep duration.

### Sampling

The sampling approach is very similar across countries as has been detailed elsewhere [28]. The sampling approach for the Peruvian cohort is broadly explained, as further analyses were conducted with this dataset. A sentinel site sampling approach was followed. The sampling strategy to choose the sentinel sites followed a multi-stage, cluster-stratified, random sampling technique. The initial sample frame at the district level was used to choose the 20 sentinel sites. In order to oversample poor areas, the top 5 percent richest districts were excluded; and poverty level was determined by the Peruvian National Fund for Development and Social Compensation. Once the districts were chosen, maps of census tracts provided by the Peruvian National Institute of Statistics and Informatics were used to randomly choose one census tract in each district, for this a random number table was used; all blocks of houses and set of houses in the selected census tract were counted, and using a random number table, one block of houses or set of houses was randomly selected per district. Finally, all households in any given block of houses or set of houses were visited to identify one with at least one child aged appropriately for the study purposes; neighboring blocks of houses or set of houses were approached until the total eligible households were found.

### Variables

The outcomes of interest were overweight and obesity assessed by BMI (Kg/m^2^). BMI was categorized into underweight, normal weight, overweight and obesity, using age- and sex-specific cut-off points proposed by Cole et al. on behalf of the International Obesity Task Force [29]. Children height and weight was measured in the three rounds [27].

The exposure variable was sleep duration assessed using the question: *How many hours does NAME sleep on a typical night?* For analysis purposes, information was categorized into short sleep (<10 hours), regular sleep (10–11 hours), and long sleep (>11 hours); as per National Sleep Foundation recommended cut-offs specific for the corresponding age range [30].

Co-variables were age (7 or 8 years old); sex (male or female); location of residence (urban or rural); child birth weight (<2500gr, 2,500–3,999gr, ≥4,000gr); number of meals the child had the previous day (<5, 5, or >5 assuming three main meals and two snacks); physical activity (set into tertiles); maternal and paternal education (none/primary school, high school, higher education); current maternal weight in kilograms (in tertiles); and wealth index (as continues and in tertiles). Some variables were included from previous rounds: maternal and paternal education from the second round and child birth weight from the first round. Table S1 shows the corresponding questions or descriptions as indicated in each country's data dictionary.

### Statistical Analysis

The analysis was conducted with STATA 11.0 (StataCorp, College Station, TX, USA). A p-value <0.05 was considered significant. Descriptive analyses were performed using Chi-square test; also, one-way analysis of variance was conducted for numerical variables. Percentages, means and standard deviations are presented. A lineal regression was calculated whit both the outcome –BMI– and exposure –sleeping hours– as numerical variables; two models were constructed: crude and adjusted by child gender, age, birth weight, total meals he/she had the previous day, physical activity, maternal and paternal education, wealth index as well as location. Also, a generalized lineal model was fitted using robust standard errors to account for the cluster effect and prevalence ratios (PR) with 95% confidence intervals (95%CI) were calculated, with exploration of effect modification by gender.

Four models were constructed to assess the association between short sleep duration and overweight or obesity: Model A, a crude exposure-outcome estimation; Model B, as before plus adding children-related variables only: age, birth weight, total meals he/she had the previous day, physical activity; Model C, as Model A plus family-related variables only: maternal and paternal education, current mother's weight, location and wealth index; and Model D, including all potential confounders considered in previous models. All models used normal weight children as the reference group. This hierarchical approach was based on recommendations from Victora et al. [31] to disentangle the effect of different levels of variables, individual and family level, in the association between short sleep duration and overweight or obesity.

### Ethics

The Young Live Study had ethical approval from the ethics committee of Social Science division, University of Oxford in the year 2006, this approval was for the study as a whole. Furthermore, ethical approval was also obtained from local ethics committees. For example, the study fostered the development of and ethics committee in Vietnam, and approval was obtained from the Research Ethics Committee at the *Instituto de Investigación Nutricional in Peru*. Each country team must have obtained informed consent of parents or caregivers; moreover, when the child had the capacity to consent, an informed consent should be obtain too. Fieldworkers were concerned to ensure that he participants had fully understood the purpose of the study, and that both parents and children fully understand what they were agreeing to; also, fieldworkers were trained to understand that informed consent must be freely given and voluntary, and that participants needed time to think about their participation. Further details on the ethics procedures have been published elsewhere (http://goo.gl/l7oAT9) and are available online (http://goo.gl/ndYtzK). To conduct this secondary data analysis ethical approval was obtained from the Institutional Review Board of Universidad Peruana Cayetano Heredia (UPCH) in Lima, Peru.

## Results

Mean age of children in Ethiopia, India, Peru and Vietnam was 8.1 (±0.3), 8.0 (±0.3), 7.9 (±0.3) and 8.0 (±0.3), respectively. Mean birth weight (grams) in Ethiopia was 3,152.1 (±781.6), 2,767.1 (±549.3) in India, 3,202.8 (±509.0) in Peru and 3,099.6 (±446.6) in Vietnam. Mean wealth index was similar among these nations: 0.3 (±0.2), 0.5 (±0.2), 0.5 (±0.2) and 0.6 (±0.2) for Ethiopia, India, Peru and Vietnam, respectively.

**Figure 1:**
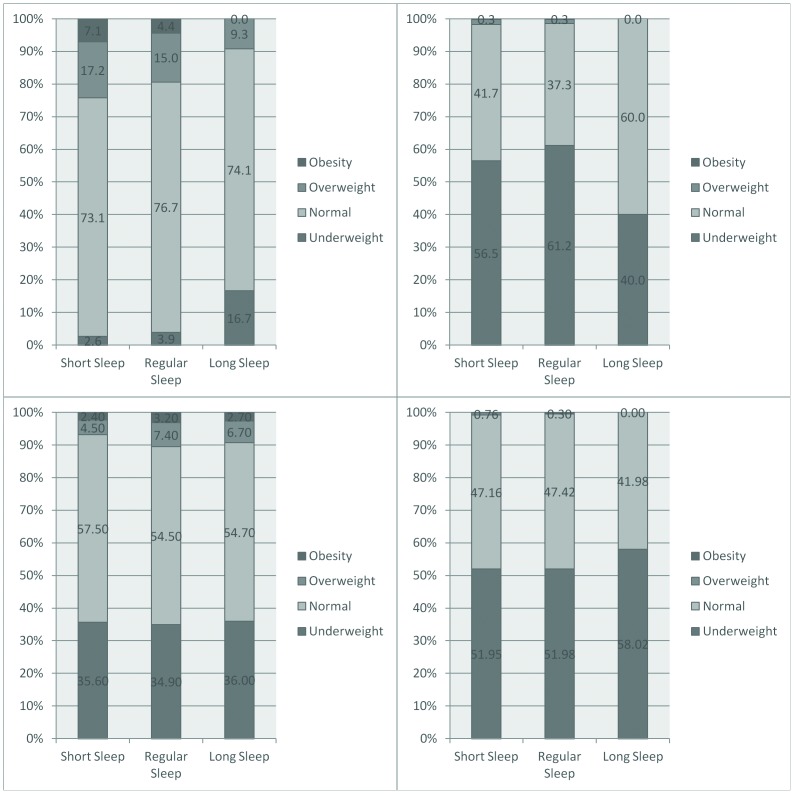
Nutritional status by sleep duration. Young Lives Study, 3° round younger cohort. Top left: Peru, Top right: India, Bottom left: Vietnam, Bottom right: Ethiopia.

The prevalence of obesity in Ethiopia and India was below 0.5%; on the other hand, the obesity prevalence was 2.8% in Vietnam and 5.4% in Peru. Underweight was highly prevalent (>50%) in India and Ethiopia, rather prevalent in Vietnam (35.2%), and only 3.7% in Peru. Sleep duration showed different patterns across these countries: the proportion of short sleep duration ranged from 41.6% in Peru to 63.5% in India (Table 1). In Peru there were a higher proportion of overweight and obese children in the short-sleep group, likewise in Ethiopia; however in Vietnam these children were mostly in the regular-sleep group (Figure 1). Given these profiles, of low prevalence of the outcomes of interest, multivariable analyses were only pursued using Peruvian data, where sleep duration was similar across genders yet BMI was higher among boys in comparison to girls (Figure S1).

**Table 1:** Nutritional status (BMI, Kg/m2) and sleep duration (hours) by country.

Variable	Peru (n = 1,929) % (95% CI)	India (n = 1,929) % (95% CI)	Vietnam (n = 1,910) % (95% CI)	Ethiopia (n = 1,882) % (95% CI)
Nutritional Status				
BMI Mean (±SD)	16.9 (2.3)	13.9 (1.6)	15.1 (2.9)	14.1 (1.4)
Underweight	3.7 (2.9–4.6)	58.1 (55.9–60.3)	35.2 (33.1–37.4)	52.2 (50.0–54.5)
Normal weight	75.1 (73.2–77.1)	40.2 (38.0–42.4)	55.8 (53.4–58.0)	47.1 (44.8–49.3)
Overweight	15.8 (14.2–17.4)	1.3 (0.8–1.9)	6.1 (5.0–7.2)	0.5 (0.2–0.8)
Obesity	5.4 (4.4–6.4)	0.3 (0.0–0.6)	2.8 (2.1–3.6)	0.2 (0.0–0.4)
Sleep Duration				
Mean (±SD)	9.6 (0.9)	9.1 (0.9)	9.7 (1.0)	9.7 (1.0)
Short	41.6 (39.4–43.8)	63.5 (61.4–65.7)	43.0 (40.8–45.2)	42.1 (39.9–44.4)
Regular	55.6 (53.4–57.8)	36.0 (33.8–38.1)	53.1 (50.8–55.3)	53.6 (51.3–55.8)
Long	2.8 (2.1–3.5)	0.5 (0.2–0.8)	3.9 (3.1–4.8)	4.3 (3.4–5.2)

Young Lives Study, 3° round younger cohort.

SD, standard deviation.

In Peru, more than half the children reported sleeping between 10–11 hours; and 41.6% slept <10 hours on an average night. Table 2 shows distribution of participants' characteristics by categories of nutritional status. Data from children with underweight was excluded from multivariable analyses.

**Table 2:** Characteristics of the participants by nutritional status.

Variables	Normal Weight (%)	Overweight (%)	Obesity (%)	Underweight (%)	P
Sleep Duration (hours)					
Mean (±SD)	9.7 (0.9)	9.5 (0.9)	9.3 (0.9)	10.1 (1.1)	<0.001**
<10 Hours	73.1	17.2	7.1	2.6	<0.001
10 to 11	76.7	15.0	4.4	3.9	
>11 Hours	74.1	9.3	0.0	16.7	
Sex	n = 1,449	n = 304	n = 104	n = 72	
Male	75.4	15.8	5.9	3.0	0.288
Female	74.8	15.8	4.9	4.5	
Age (years)	n = 1,449	n = 304	n = 104	n = 72	
Mean (±SD)	7.9 (0.3)	7.9 (0.3)	7.9 (0.3)	7.9 (0.3)	0.125**
7 years	74.1	17.3	5.6	3.0	0.077
8 years	76.3	14.0	5.1	4.6	
Birth Weight (gr)	n = 1,238	n = 284	n = 104	n = 64	
Mean (±SD)	3171.6 (504.3)	3309 (481.5)	3389 (558.8)	3028 (492.3)	<0.001**
≤2500 gr	72.3	10.6	7.5	9.6	0.013
2,500–4,000 gr	73.6	17.2	5.7	3.5	
≥4,000 gr	68.4	17.4	11.2	3.1	
Number of meals	n = 1,447	n = 304	n = 103	n = 72	
Mean (±SD)	4.8 (0.9)	5.0 (1.0)	5.1 (0.8)	4.8 (0.9)	0.002**
<5	79.1	13.2	4.1	3.7	0.014
5	75.3	15.3	5.4	4.0	
>5	69.3	20.4	7.1	3.2	
Physical Activity (days)	n = 1,444	n = 302	n = 103	n = 72	
Mean (±SD)	3.7 (2.7)	3.6 (2.7)	2.2 (2.1)	4.2 (2.5)	<0.001**
Bottom	73.2	15.9	8.2	2.8	<0.001
Middle	73.0	16.0	5.3	5.8	
Top	79.4	15.3	1.4	3.9	
Location	n = 1,449	n = 304	n = 104	n = 72	
Urban	70.3	18.7	7.4	3.6	<0.001
Rural	87.2	8.4	0.4	4.0	
Maternal Education	n = 1,419	n = 298	n = 101	n = 71	
None/Primary	83.4	10.6	1.1	5.0	<0.001
High School	72.4	16.6	7.7	3.3	
Higher	60.3	26.7	11.2	1.7	
Paternal Education	n = 1,383	n = 294	n = 98	n = 69	
None/Primary	83.2	10.6	1.6	4.6	<0.001
High School	74.0	16.9	5.7	3.4	
Higher	63.9	22.6	10.5	3.1	
Maternal Weight (Kg)	n = 1,388	n = 289	n = 101	n = 65	
Mean (±SD)	59.7 (9.9)	65.2 (11.7)	68.4 (11..8)	55.7 (10.6)	<0.001**
Bottom	83.1	10.2	1.6	5.0	<0.001
Middle	76.1	15.3	5.5	3.1	
Top	66.6	21.7	9.4	2.3	
Wealth Index	n = 1,447	n = 304	n = 103	n = 72	
Bottom	86.8	8.1	0.8	4.4	<0.001
Middle	76.7	15.0	4.2	4.0	
Top	61.9	24.2	11.1	2.8	

Young Lives Study, 3° round Peruvian younger cohort.*

*Some co-variables have missing values.

**One-way analysis of variance test.

### Association between short sleep and overweight or obesity

In the simple lineal regression, including both boys and girls, there was a decrease in BMI units per each additional hour sleeping: β = −0.33 (95%CI: −0.44; −0.23) and β = −0.19 (95%CI: −0.31; −0.07) in the crude and adjusted model, respectively (Supplementary Table 2).

**Figure 2:**
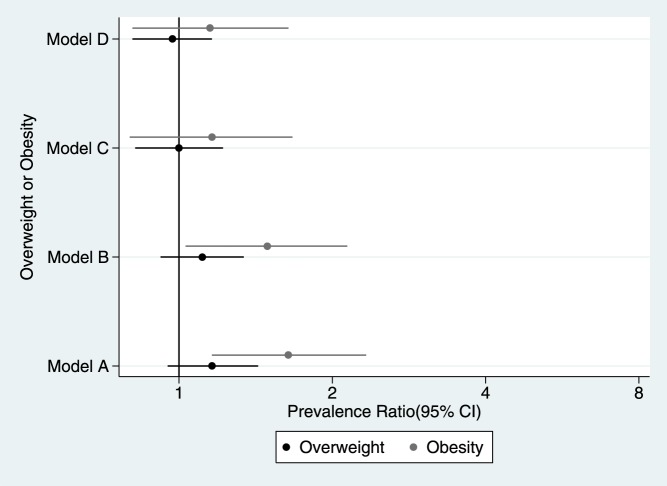
Prevalence ratio of overweight and obesity. Young Lives Study, 3° round younger cohort. Model A: crude exposure-outcome estimation. Model B: only adjusted for age, birth weight, total meals previous day and physical activity. Model C: only adjusted for maternal education, paternal education, maternal weight, location and wealth index. Model D: adjusted for all the co-variables.

**Figure 3:**
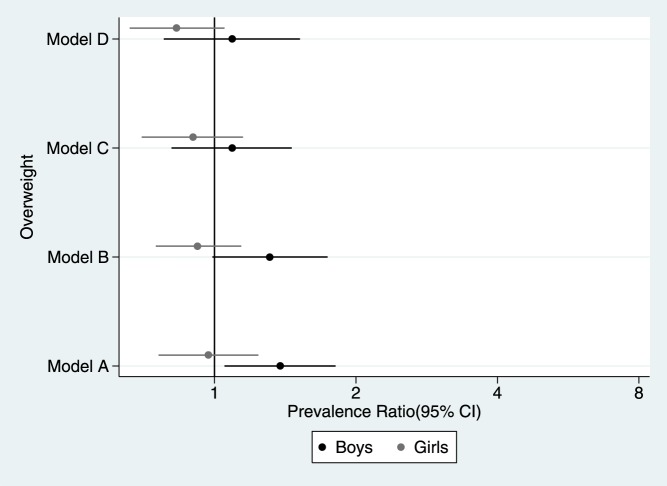
Prevalence ratio of overweight according to child gender. Young Lives Study, 3° round younger cohort. Model A: crude exposure-outcome estimation. Model B: only adjusted for age, birth weight, total meals previous day and physical activity. Model C: only adjusted for maternal education, paternal education, maternal weight, location and wealth index. Model D: adjusted for all the co-variables.

When including both boys and girls there was no evidence of an association between short sleep duration and overweight in any of the models (Figure 2). Sex was an effect modifier of the relationship between short sleep duration and overweight (p = 0.030), but not of the association between short sleep duration and obesity (p = 0.533). There was a trend for girls with sleeping patterns of less than ten hours per night to have lower prevalence of overweight; there was the opposite trend for boys: higher prevalence of overweight (Figure 3).

In the fully-adjusted model, there was a trend for children sleeping <10 hours to have more chances of being obese compared to those sleeping 10 to 11 hours (Figure 2). Exact details on the regression models and its estimates are shown in Table 3. Each child-associated variable slightly attenuated the association between short sleep duration and obesity, and family-related variables showed a stronger attenuation; all regressions were statistical significant except when the confounders were maternal education and wealth index (Table 4).

**Table 3:** Association between short sleep duration and overnutrition.

Sleep Duration	Model A	Model B	Model C	Model D
	PR (95%CI)	PR (95%CI)	PR (95%CI)	PR (95%CI)
Overweight				
	n = 1,708	n = 1,480	n = 1,562	n = 1,361
10–11 hours	1	1	1	1
<10 hours	1.16 (0.95–1.43)	1.11 (0.92–1.34)	1.00 (0.82–1.22)	0.97 (0.81–1.16)
Overweight Boys				
	n = 871	n = 766	n = 796	n = 701
10–11 hours	1	1	1	1
<10 hours	**1.38 (1.05**–**1.81)**	1.31 (0.99–1.74)	1.09 (0.81–1.46)	1.09 (0.78–1.52)
Overweight Girls				
	n = 837	n = 714	n = 766	n = 660
10–11 hours	1	1	1	1
<10 hours	0.97 (0.76–1.24)	0.92 (0.75–1.14)	0.90 (0.70–1.15)	0.83 (0.66–1.05)
Obesity*				
	n = 1,513	n = 1,306	n = 1,385	n = 1,204
10–11 hours	1	1	1	1
<10 hours	**1.64 (1.16**–**2.33)**	**1.49 (1.03**–**2.14)**	1.16 (0.80–1.67)	1.15 (0.81–1.64)

Young Lives Study, 3° round Peruvian younger cohort.

Model A: crude exposure-outcome estimation.

Model B: only adjusted for age, birth weight, total meals previous day and physical activity.

Model C: only adjusted for maternal education, paternal education, maternal weight, location and wealth index.

Model D: adjusted for all the co-variables.

*For obesity, model B included the child gender and so did model D.

Results in bold are statistical significant. PR: prevalence ratio. 95%CI: 95% confidence interval.

**Table 4:** Association between short sleep duration and obesity adjusted by each confounder at a time.

Model	PR (95% CI)
	Overall
Crude	**1.64 (1.16**–**2.33)**
Child-related variables	
Adjusted by child age*	**1.64 (1.16**–**2.32)**
Adjusted by child gender	**1.65 (1.16**–**2.34)**
Adjusted by birth weight	**1.59 (1.34**–**2.23)**
Adjusted by number of meals	**1.56 (1.11**–**2.20)**
Adjusted by physical activity	**1.59 (1.09**–**2.31)**
Family-related variables	
Adjusted by maternal education	1.35 (0.94–1.92)
Adjusted by paternal education	**1.45 (1.00**–**2.11)**
Adjusted by maternal weight	**1.40 (1.06**–**1.86)**
Adjusted by location	**1.47 (1.06**–**2.06)**
Adjusted by wealth index	1.29 (0.92–1.81)

Peruvian younger cohort.

*Adjusted only by child age.

Results in bold are statistical significant. PR: prevalence ratio. 95%CI: 95% confidence interval.

## Discussion

### Main Findings

Across four different developing countries, the prevalence of childhood obesity was very much dissimilar, ranging from 0.2% in Ethiopia to 5.4% in Peru. This calls for a continuing monitoring of childhood obesity trends in developing countries. In the sample of Peruvian school-aged children, there was a trend for the association between short sleep duration and obesity: those who slept less than 10 hours have a 15% greater prevalence of obesity when compared to children who sleep between 10 to 11 hours after adjusting for several potential confounders using a hierarchical strategy for adjustment. Gender was an effect modifier of the association between short sleep duration and overweight but not with obesity; the latter observation should be taken with caution provided the low prevalence of obesity. In fact, the opposite expected pattern, girls with short sleep duration seemingly having lower chances of being overweight, was observed.

Although the association of interest was not pursued with data from Ethiopia, India and Vietnam, these settings should be careful regarding sleep duration in the children population as it seems to be already short and children from a low-income background are at higher risk of insufficient sleep [32]; moreover, a study conducted with Asian children -including India and Vietnam- reported short sleep duration [33]. The authors believe it is worth showing results from the four countries to emphasize the different patterns in childhood obesity and the prevalence of short sleep duration.

### Hierarchical Approach

Family-related variables attenuated the association between short sleep and obesity. These variables may play an important role in the proposed association; children at these ages still depend on their parents and surrounding environment.

Maternal educational and wealth index attenuated the most the association between short sleep duration and obesity. In the Peruvian sample, parents with none/primary education were mostly in the bottom wealth index tertile (data not shown); consequently, issues associated with poverty may play a role in this association. For example, El-Sheikh *et al.* reported that children in households with a lower income-to-needs ratio have higher levels of sleep problems [32]. Housing and overcrowding could be a concern: many family members may need to share one bedroom. This could lead to inadequate sleep duration or quality, as it has been reported that children who sleep with their parents have sleep curtailment [13]. Also, we hypothesize that parents with low educational attainment and conducting households with low wealth index may need to have more than one job, this could lead to less sleep hours; unfortunately, this habit could be adopted by their children. Jiang et al. found that preschool children, whose caregivers sleep less, also had short sleep duration [13]. Furthermore, the effect of socioeconomic status could be greater than our results show because there is a residual confounding as the sampling procedure excluded the 5% richest districts.

### Comparison with previous studies

Some of the studies compiled in systematic reviews and meta-analysis have also failed to find evidence of the association between short sleep duration and overweight and obesity [22], [23], [34]. Our results could be explained by the low prevalence of both overweight (15.8%) and obesity (5.4%), in comparison to other settings where similar studies had been conducted, and to our short sleep duration definition which is much conservative than those used in other studies. For example, Jiang *et al.* found a prevalence of obesity in boys and girls of 10.3% and 6.9% respectively, and their strongest odds ratio was found when comparing children sleeping <9 hours versus the reference sleep duration (>11 hours); when they compared their reference against children sleeping 10 or 9.5 hours no significant results were reported [13]. Likewise, a study with Australian children reported an obesity prevalence of 7.7%, and they showed a strong association when comparing children sleeping <9 hours, versus those sleeping >10 hours; though their results were not significant when comparing against those sleeping 9–10 hours [10]. In Quebec they found a prevalence of overweight/obesity of 22%; however, they did find a strong association when comparing children sleeping 10.5–11.5 hours with those sleeping 12–13 hours [12]. Systematic reviews and meta-analysis have included studies that compared short sleep duration versus a reference of ≥10 hours, reaching significant results; nevertheless, it is worth noting that children from developing countries were underrepresented [23], [35].

### Boys and girls: the association between short sleep duration and overweight

Some authors have postulated the association between short sleep duration and overweight or obesity is present in boys but not in girls [10], [14]. It has been suggested that females cope better with environmental stress, and thus they would need a much lower sleep duration to put them at risk of overweight or obesity [14], [36]. Eisenmann *et al.* postulated a similar hypothesis, finding a higher BMI among children in a new short sleep duration category (<6 hours), previously their shortest sleep duration category was <8 hours; fortunately for their analysis they managed to find girls sleeping <6 hours [14]. We were prevented from conducting a similar post-hoc analysis because in the Peruvian sample there were only 21 girls sleeping less than eight hours. Depending on the context, cut-offs used for (short) sleep duration may vary for boys and girls, to observe likelihood of overnutrition. As long as urbanization, acculturation and changes in sociodemographic profiles foster a shift towards short sleep duration, children in developing countries may need to be addressed to prevent the associated risk towards overweight and obesity.

### Strengths & Limitations

The strengths of this study include a large sample size, the inclusion of data of four developing settings located in different world regions, and the assessment of several potential confounders, as well as a hierarchical approach which enabled us to study the confounding effect of variables at different levels. Also, the use of sleep duration cut-off points based upon recommendations of a well-known organization. However, there are limitations to be addressed. First, the cross-sectional design prevents us from determining causality; also, reverse causation could be an issue. Nevertheless, longitudinal studies have supported the hypothesis that short sleep duration is a risk factor for overweight and obesity. Second, children sleep duration was assessed through parental report; however, this could have affected very little the results because there is a good correlation between the subjective and objective sleep duration as per parental [37] or children report [38]. Third, data on sleep duration was retrieved for a single night, assessing this variable for different nights throughout the week or in repeated assessments could lead to a better understanding of sleep restriction; especially as stable sleep duration throughout the week might make the children less likely to develop metabolic misbalance [39] and compensatory sleep during weekends may ameliorate the risk of higher BMI [40]. Fourth, although we included several potential confounders, such as physical activity and diet, these were not objectively assessed; for instance, physical activity was based upon mother's report which could have been over- or underestimated. However, we believe these variables were fine proxies to be included in the models; in addition, some studies [6], [8], [10], [12], [13], [17]–[19] have included these confounders assessed in a similar fashion, and others have not included them at all [14], [16].

Future studies will benefit from a randomized trial design to draw a firm conclusion on whether sleep duration is a valid venue to prevent childhood obesity, or if it should at least be included along with other prevention strategies. To our knowledge there are a few randomized studies registered on this subject (http://goo.gl/Wv7OJn); their results should give much clear evidence on the association between short sleep duration and childhood obesity.

## Conclusions

Childhood overweight and obesity have different profiles across developing settings; though sleep duration is rather similar. In a sample of children living in resource-limited settings in Peru, a country in epidemiological transition, there is no association between short sleep duration and obesity; interestingly, the crude association was slightly attenuated by children-related variables but strongly diminished by family-related variables such as parental education and wealth index. The findings are of special interest given the growing prevalence of childhood overweight and obesity in developing countries, and its associated health and societal burdens. Given the rising prevalence of childhood obesity worldwide, sleep duration could be considered as an additional avenue to introduce obesity prevention strategies.

## Supporting Information

Figure S1 **Box-plot of sleep duration (hours) and BMI (Kg/m2) according to child gender in Peru.** Young Lives Study, 3° round younger cohort.
(DOCX)

Table S1 **Questions and descriptions posed in each country's data dictionary to collect information on variables included in this study.**
(DOC)

Table S2 **Lineal regression between sleep duration (hours) and BMI (Kg/m2).** Young Lives Study, 3° round younger cohort, Peru.
(DOCX)
