# Supplementary material for: Short Sleep Duration and Childhood Obesity: Cross-Sectional Analysis in Peru and Patterns in Four Developing Countries
Source: PLoS One. 2014 Nov 13;9(11):e112433. doi: 10.1371/journal.pone.0112433 (PMC4231052; doi:10.1371/journal.pone.0112433)
Supplement: Figure S1 — Box-plot of sleep duration (hours) and BMI (Kg/m2) according to child gender in Peru. Young Lives Study, 3° round younger cohort. (DOCX) [file pone.0112433.s001.docx]

Figure S1: Box-plot of sleep duration (hours) and BMI (Kg/m2) according to child gender in Peru. Young Lives Study, 3° round younger cohort.
