# Supplementary material for: Short Sleep Duration and Childhood Obesity: Cross-Sectional Analysis in Peru and Patterns in Four Developing Countries
Source: PLoS One. 2014 Nov 13;9(11):e112433. doi: 10.1371/journal.pone.0112433 (PMC4231052; doi:10.1371/journal.pone.0112433)
Supplement: Table S1 — Questions and descriptions posed in each country's data dictionary to collect information on variables included in this study. (DOC) [file pone.0112433.s002.doc]

Table S1

| **Variable** | **Question/description** | | | |
| --- | --- | --- | --- | --- |
|  | **Peru** | **India** | **Vietnam** | **Ethiopia** |
| BMI | Calculated bmi=weight/squared(height) | Calculated bmi=weight/squared(height) | Calculated bmi=weight/squared(height) | Calculated bmi=weight/squared(height) |
| Sleep duration | How many hours does NAME sleep on a typical night? | Hours per day – sleeping | How many hours does NAME spend asleep in a typical night? | Hours sleep in a typical night. |
| Birth weight | Birth weight (in grams) | Birth weight (in grams) | Birth weight (in grams) | Birth weight (in grams) |
| Number of meals | Total times NAME ate something in last 24 hrs. | Total times child ate in the last 24 hrs. | Total times NAME are something in the previous 24hr period. | Total times NAME ate in the last 24 hours. |
| Physical activity | During the last 7 days on how many days was NAME physically active for at least 60 min. at one time? | In the last 7 days on how many days was NAME physically active for at least 60 minutes? | Not available | In the last 7 days on how many days was NAME physically active for at least 60 minutes? |
| Maternal/Paternal education | What is the highest education grade completed by the mother (excluding pre-primary)? Same question for the father. | What is the highest education grade completed by the mother (excluding pre-primary)? Same question for the father. | What is the highest education grade completed by the mother (excluding pre-primary)? Same question for the father. | What is the highest education grade completed by the mother (excluding pre-primary)? Same question for the father. |
| Maternal weight | Agreed maternal weight. | Agreed maternal weight. | Agreed maternal weight. | Agreed maternal weight. |
| Location | Is this an urban or rural site. | Urban or rural | No question on dictionary for round three. | Urban or rural |
| Wealth index | Composite variables – wealth index | Composite variables – wealth index | Composite variables – wealth index | Composite variables – wealth index |
