# Supplementary material for: Short Sleep Duration and Childhood Obesity: Cross-Sectional Analysis in Peru and Patterns in Four Developing Countries
Source: PLoS One. 2014 Nov 13;9(11):e112433. doi: 10.1371/journal.pone.0112433 (PMC4231052; doi:10.1371/journal.pone.0112433)
Supplement: Table S2 — Lineal regression between sleep duration (hours) and BMI (Kg/m2). Young Lives Study, 3° round younger cohort, Peru. (DOCX) [file pone.0112433.s003.docx]

Table S2

|  | Crude Model  N=1,929  Β (95%CI) | Adjusted Model*  N=1,545  Β (95%CI) |
| --- | --- | --- |
| Sleep duration (hours) | -0.33 (95%CI: -0.44;-0.23) | -0.19 (95%CI: -0.31;-0.07) |

*Adjusted by adjusted by child gender, age, birth weight, total meals he/she had the previous day, physical activity, maternal and paternal education, wealth index as well as location.
